# Maximizing the impact of HIV prevention technologies in sub‐Saharan Africa

**DOI:** 10.1002/jia2.25319

**Published:** 2019-07-22

**Authors:** Helen Ward, Geoffrey P Garnett, Kenneth H Mayer, Gina A Dallabetta

**Affiliations:** ^1^ Infectious Disease Epidemiology Imperial College London London United Kingdom; ^2^ Bill & Melinda Gates Foundation Seattle WA USA; ^3^ The Fenway Institute Harvard Medical School Boston MA USA

**Keywords:** HIV, prevention, pre‐exposure prophylaxis, Africa South of the Sahara, health technology

## 1 Introduction

There have been substantial gains in the range and efficacy of technologies available for HIV prevention, with voluntary medical male circumcision (VMMC), treatment as prevention, and pre‐exposure prophylaxis (PrEP) being added to the existing toolbox of condoms, lubricant, behaviour change, harm reduction, structural interventions and advocacy programmes. These advances led to the optimism of calls to end the AIDS epidemic by 2030 with a target of fewer than 500,000 new cases a year by 2020 and 200,000 by 2030 1, 2. However, progress towards these goals is slow with an estimated 1.9 million new infections in 2017 globally, including over a million in sub‐Saharan Africa 3. Although these numbers represent a substantial reduction from the height of the epidemic in the late 1990s, they are well off target despite intensive efforts to promote HIV combination prevention encompassing structural, behavioural and biomedical interventions. The targets were based on modelling which estimated the coverage needed to achieve these reductions, namely meeting 90‐90‐90 treatment targets, 90% coverage of key populations with combination prevention programmes, 90% reported condom use rates with non‐regular partners and 90% male circumcision 2. However, investment in prevention has been lower than required to achieve this coverage 4, and results from trials of intensive population “test and treat” approaches show a lower impact than hoped for, with up to 30% reduction in incidence, but with lower engagement and coverage of younger people 5, 6.

Reducing incident infections is a key to sustainable HIV control, and epidemic models suggest that further primary prevention is necessary in addition to treatment for those already infected if the 2030 targets are to be reached 7. This all points to the need for increased efforts in order to maximize the impact of technologies, including those currently in development. While there is good evidence for efficacy of the technologies and strategies in some populations 8, 9, they have failed to reach their potential in many high incidence populations in sub‐Saharan Africa 10. In particular, the lack of widely available efficacious female‐controlled methods has left many young women vulnerable to HIV when their partners are unwilling to use condoms 11. While newer technologies such as long‐acting injectable PrEP have potential for additional impact, they too may disappoint if the lessons of existing programmes are not learned and programmes not scaled sufficiently.

Adolescent girls and young women are at particular risk, accounting for 25% of new HIV infections among adults in sub‐Saharan Africa, and with three to five times the prevalence of adolescent boys and young men 7, 10. Reducing the high incidence in these young populations is perhaps the most urgent global challenge, particularly as 60% of the population in sub‐Saharan Africa is aged under 25 and absolute numbers of young people will continue to rise over the coming decades 12.

It will be important to identify the mix of prevention technologies and approaches that will meet the needs of these young people, in order to design the interventions that will support their adoption. This requires an interdisciplinary approach, with the appropriate mix of social, behavioural, epidemiological and programme science, with learning from key stakeholders including young people themselves, service providers, policy makers, industry and governments. The community perspectives about health and HIV, and their health seeking behaviour need to be taken into account along with the determinants of decisions about the HIV risk behaviours and the use of HIV prevention technologies. Demographic, geographic and other characteristics should inform how HIV prevention interventions are prioritized and should be designed around the needs and preferences of priority users.

## 2 The technologies

Condoms provide triple protection against HIV, pregnancy and many other sexually transmitted infections 13. They are often easily accessible, inexpensive and when carried provide an option for unplanned sexual activity. Condoms have been promoted over the course of the HIV pandemic with overall use increasing, reducing incidence among key populations (men who have sex with men (MSM) and sex workers) where sexual partnerships can be brief 14, 15. One important lesson from condom programmes in the general population is that marketing and promotion are essential for generating demand. However, funding for condom promotion and marketing has decreased, particularly since 2011, often leading to condom supplies sitting unused due to lack of demand 16. The lack of demand may reflect problems of access as distribution programmes have been cut and condoms may be less widely available outside of health facilities in some countries. Perhaps the most important lesson from male condoms is that an intervention for women that requires partners to change their behaviour may be an insurmountable barrier to use in many contexts, exacerbated by the perceptions that condoms decrease sexual pleasure and may undermine trust in relationships.

Oral PrEP using tenofovir disoproxil fumarate and emtricitabine (TDF/FTC) is an efficacious HIV prevention product. In developed countries oral PrEP use is reducing HIV incidence among MSM 17. However, there are important barriers to its uptake and use: it is relatively costly, so it can be difficult to access; it is specific for prevention and treatment of HIV so its uptake and use can be stigmatized; it may have side effects when first taken; it requires daily dosing to be effective for women; and it requires medical monitoring for side effects and breakthrough infections 18. The introduction of oral PrEP and its scale‐up has been slow for many reasons, but we are now in a position where oral PrEP is being introduced for populations of MSM, sex workers, those in HIV sero‐discordant couples, and young women in a small number of locations in sub‐Saharan Africa. We are just starting to learn from oral PrEP programmes for young women and there will be much to learn from their evaluation. Many future potential interventions (such as cabotegravir injections, broadly neutralizing antibody [bNAb] injections, ARV implants or vaccines) will not have daily use requirements, but will share many of the same barriers to uptake as oral PrEP 19.

VMMC is another efficacious intervention and scale‐up across sub‐Saharan Africa has been ongoing for several years with mixed effect, but over four million men were circumcised in 2017 alone, and the programme has averted an estimated 230,000 HIV infections. Comprehensive programmes including HIV testing and counselling, safer sex education and condom promotion and distribution 20.

## 3 The HIV prevention cascade

**Figure 1:**
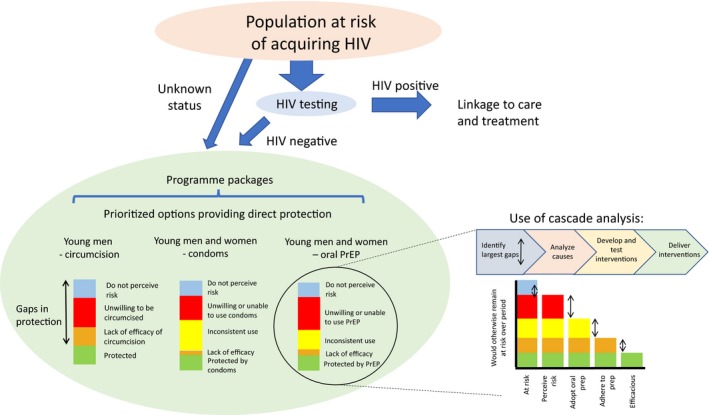
Example of use of HIV Prevention Cascades at Programme Level. Diagram starts with population at risk of HIV, identifies those who would benefit from a primary prevention intervention package, and for each intervention identifies gaps in protection. For one intervention, oral PrEP, the right side of the diagram suggests how a cascade analysis can identify and address the largest gaps.

HIV prevention programmes are complex to design, deliver and evaluate, and an HIV prevention cascade has been suggested as a tool to support prevention programming 21. Such cascades identify key steps for interventions to be effective, including identification of populations at risk, perception of risk, intervention uptake and use, and ultimately the efficacy of the intervention. Once the cascade steps are identified and quantified, programme planners can see where the largest gaps lie and take steps to understand and address them. Figure 1 shows how prevention cascades may be used at the programme level, starting from a population at risk and identifying who would benefit from primary prevention packages, that is, those who test HIV negative or who do not know their status. The options illustrated in the figure include VMMC for young men, and condoms or oral PrEP for young men and women, and the cascades show the potential gaps in protection.

For example, defining who is at risk will depend on local HIV prevalence and patterns of risk within the community. There has been relatively little recent work measuring patterns of risk in young women, but in Southern and East Africa the risks include transactional sex (broadly defined), age‐disparate partnerships, multiple sex partners, alcohol use, being or having partners uncircumcised, partners who travel, and for young women risk seems to increase with time after sexual debut and be associated with marriage 22, 23, 24, 25. The first step of the cascade is perception of risk, and studies of young women show that perceived risk of HIV acquisition is often lower than actual risk. In various studies in sub‐Saharan Africa, between 17% and 50% of women deemed to be at high risk of incident infection perceived themselves to be at risk, while another study showed little association with self‐perception of HIV risk and subsequent acquisition of infection 26. Understanding more about this mismatch between risk and risk perception will be crucial to developing interventions to drive demand and uptake for effective interventions. The next step would be for an individual (and sometimes their partner) to take up and use an intervention. Many factors influence such decisions, and the decisions have to be made and acted upon repeatedly in interventions such as condoms, sexual behaviour change or PrEP use. Consistent use is a particular challenge of HIV prevention: because there is low risk in most partnerships and high risk in a small fraction, HIV prevention interventions have to provide protection across all sex acts within those high‐risk partnerships for their benefits to be realized 27.

## 4 Social epidemiological framework

The prevention cascade can be used alongside a social epidemiological framework which highlights the influence of structural factors, such as laws, policies, regulations, relational factors, such as family, relationship status, economic situation and more immediate factors, such as setting and privacy, intimate partner violence, alcohol and drug use 28, 29. These factors will interact with people's preferences and motivations to determine their use of any particular technology. Additional barriers to demand for the uptake and use of interventions include lack of awareness, lack of self‐efficacy, difficulty in accessing services, stigma associated with uptake (e.g. by health‐care workers), stigma of use (e.g. by partners), difficulty of use and side effects of use.

We can of course learn from examples where programmes have been effective. Reproductive health programmes promoting access to contraception have been extremely successful in many settings, despite requiring many similar behaviours and in similar contexts to HIV prevention. Their successes have been put down to many factors, but key to them are leadership and effective management, appropriate communication strategies, evidence‐based programming, high quality contraceptive methods, availability, trained staff, client‐centred care, choice of methods, access and variety of outlets, affordable, involvement of men and women, and integration with other services 30, 31, 32. Effective HIV prevention programmes for sex workers have also been based on community mobilization, advocacy, access to integrated HIV prevention, STI control, contraception and other services 14.

## 5 Banbury meeting and supplement

**Figure 2:**
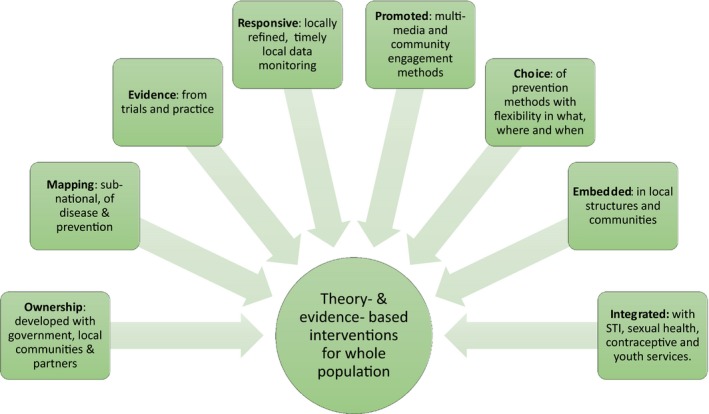
Model for maximizing HIV prevention impact at Programme level. Model developed through consensus discussion at Banbury Center Meeting, May 2017 33. The outer boxes show key characteristics and inputs of a theory‐ and evidence‐ based prevention programme which are required to deliver the holistic intervention package.

In 2017, a small meeting took place at the Banbury Center which hosts think‐tanks on key questions in molecular biology, genetics neuroscience and science policy, on how to maximize the impact of HIV prevention technologies in sub‐Saharan Africa 33; we brought together international experts from different disciplines to address how to make the development and delivery of HIV prevention technologies more successful. The meeting concluded that many elements will be needed for effective and sustained primary HIV prevention, including local ownership, community engagement and acceptability, good evidence and data to guide planning and implementation and integration of primary HIV prevention into local service prevision, including general sexual and reproductive health services (Figure 2). The presentations and discussions at that meeting led to the commissioning of this supplement with contributions addressing some of the gaps in our knowledge, and showing the potential role of disciplines ranging from mathematical modelling and social psychology to marketing and behavioural economics.

In this supplement, we have brought together a number of articles that build on some of the discussions at that meeting, representing a variety of disciplinary perspectives. Mojola and Wamoyi start with a narrative and insightful review into the drivers of HIV risk among young African women 34, and use insights from their own and others’ research to show how epidemiological, gender‐normative and environmental contexts interact to drive hyperendemics of HIV, and how similar factors undermine preventive interventions. Their deep descriptions of how social drivers result in risky settings and behaviours are then used to suggest how that context can inform intervention planning. In the next article, Skovdal draws on contemporary social theory to illustrate the need for a greater understanding of the links between the different determinants of preventive behaviours 35. He argues that through exploring social practices we can better understand these phenomena, and proposes a “table of questioning” that can be used by programme planners. The next paper is a viewpoint from Gomez and colleagues who explore the application of market segmentation to prevention programmes 36. In public health we are used to describing and grouping people by sociodemographic characteristics that predict risk or define the need for an intervention. In marketing techniques developed in the commercial sector, segmentation is based on a range of other factors, psychographic and behavioural, which are said to better predict consumer behaviours. The authors describe examples of market segmentation in public health, and argue that we would do well to adopt some of these techniques to more closely match our programming to the desires and preferences of the people we want to engage, developing segment‐specific campaigns. This resonates with the broad interest in “personalized prevention,” but it is clear that evidence is needed as to how and where such approaches are effective.

The next group of papers shift from frameworks to evidence from interventions. Celum and colleagues provide a timely and comprehensive review of the state of knowledge on pre‐exposure prophylaxis for adolescent girls and young women in Africa 37. The paper summarizes lessons from PrEP implementation projects and concludes that these are feasible interventions, but identified significant challenges; like Gomez they identify the need for appropriate and targeted messages for demand creation; the need for youth friendly and integrated services that address wider concerns such as sexually transmitted infections and contraception; the need for novel approaches to supporting adherence. Eakle and colleagues’ paper is a scoping review of the evidence on the perspectives and experience of using PrEP among people at risk of HIV in sub‐Saharan Africa 38. From 35 included papers, they were able to identify five themes which affected acceptability and utilization. These resonate with many of the factors highlighted elsewhere in this issue, including empowerment and stigma, complex risk environments and relationships as well as specifics concerning efficacy, side‐effects and practical challenges in use. This speaks to the need for more in‐depth research into user views and priorities if current and future technologies are to be widely implemented. A further review by Ensor and colleagues draws together quantitative and qualitative evidence from 18 papers on the effectiveness of demand creation interventions for male circumcision programmes 39. Financial incentives that compensate for loss of income and costs have a large relative impact on uptake, but absolute effect was larger in programmes involving community leaders and education.

Pettifor and colleagues conducted a qualitative study of a cash transfer project in Tanzania, and propose a conceptual framework for the possible mechanisms for reducing HIV risk 40. Reduced dependence on transactional sex may be one mechanism, but they also identified the importance of business education and mentorship in building young women's efficacy and self‐esteem, and argue that these may have a greater impact in the long term. This kind of qualitative research is able to explore how interventions work, an essential part of the transition from efficacy to implementation.

The final group of papers address the programme science of HIV prevention, which should provide evidence of who to target with which interventions, how to evaluate, adjust and continually strengthen programmes in response to feedback 41. Cowan and colleagues use programme data to inform a mathematical model in order to assess whether scale up of interventions for sex workers could contribute to elimination of HIV in Zimbabwe 42. They estimate that up to 70% of all new HIV infections could have been averted if sex work interventions had achieved complete coverage in 2010; while they recognize the limitations of this as a model, the paper raises an important point by showing that appropriate scaling of interventions is essential if they are to have impact at a population level. One obstacle to such scale‐up for programmes is cost, and this is addressed in the paper by Roberts and colleagues 43. Using data from a PrEP implementation project in Kenya, they identified costs of the programme and explored different scenarios to show that incremental costs were sensitive to the delivery approach and the extent to which monitoring, for example routine creatinine testing, were included. The paper provides estimates that can be used to explore the cost‐effectiveness and budget impact when providing PrEP that will be an import contribution to policy and practice with this relatively new intervention. The paper has important implications for contemporary discussions of different PrEP delivery models such as pharmacy‐based and direct to consumer marketing.

In Kenya, HIV Prevention Cascades have been used as programme management tools, and Bhattacharjee and colleagues describe their use in combination HIV prevention interventions for sex workers 44. The paper shows that the cascade, while intended as a relatively simple tool, can be quite complex in practice. The first challenge was to agree the target population or denominator, which is challenging for relatively hidden and transient populations, and then measures of uptake have to be defined. Despite these challenges, they show how such data helped identify significant gaps in the programme, and by using the same approach at a delivery level, service providers can reflect on and improve their own performance. The final paper from Moorhouse et al. describes how the HIV Prevention Cascade informed the identification and evaluation of interventions in Zimbabwe 45. They propose a standard approach to the use of the cascade to develop interventions.

## 6 Conclusions

Technological innovations hold enormous promise for improving health, and in HIV research there have been remarkable advances in the development of efficacious tools for treatment and prevention. Ensuring that these lead to maximum impact is just as much of a challenge as developing the technologies themselves. In populations with the highest HIV incidence, achieving impact will require the close collaboration of a wide range of stakeholders and disciplines, political will, investment and ongoing evaluation and programme iteration. The articles in this supplement have focused on some of the advances and insights from diverse disciplinary backgrounds. The contributions have largely focused on young women, which was an initial priority as we understand that they have often been failed by previous programmes and technologies. Now that we have female‐controlled methods the onus is on us to support women in accessing and using them. However, we recognize this focus as a limitation, with men, particularly young men in Southern and East Africa, also being at ongoing risk with limited access to the resources and methods to protect themselves and their partners.

We hope that this journal issue will spark more interest in this evolving field and contribute to the progress required to end AIDS.

## Competing interests

All authors have no competing interest to declare.

## Authors’ contributions

H.W. and G.G. drafted the manuscript, all authors contributed to revisions and approved the final version.
